# Effect of the Hypoxia Inducible Factor on Sorafenib Resistance of Hepatocellular Carcinoma

**DOI:** 10.3389/fonc.2021.641522

**Published:** 2021-07-07

**Authors:** Zhi Zeng, Qiliang Lu, Yang Liu, Junjun Zhao, Qian Zhang, Linjun Hu, Zhan Shi, Yifeng Tu, Zunqiang Xiao, Qiuran Xu, Dongsheng Huang

**Affiliations:** ^1^ The Medical College of Qingdao University, Qingdao, China; ^2^ Zhejiang Provincial People’s Hospital (People’s Hospital of Hangzhou Medical College), Hangzhou, China; ^3^ Graduate Department, Bengbu Medical College, Bengbu, China; ^4^ The Second Clinical Medical College of Zhejiang Chinese Medical University, Hangzhou, China; ^5^ The Key Laboratory of Tumor Molecular Diagnosis and Individualized Medicine of Zhejiang Province, Zhejiang Provincial People’s Hospital (People’s Hospital of Hangzhou Medical College), Hangzhou, China

**Keywords:** sorafenib, hepatocellular carcinoma, HIF-1α, HIF-2α, sorafenib-resistance

## Abstract

Sorafenib a multi-target tyrosine kinase inhibitor, is the first-line drug for treating advanced hepatocellular carcinoma (HCC). Mechanistically, it suppresses tumor angiogenesis, cell proliferation and promotes apoptosis. Although sorafenib effectively prolongs median survival rates of patients with advanced HCC, its efficacy is limited by drug resistance in some patients. In HCC, this resistance is attributed to multiple complex mechanisms. Previous clinical data has shown that HIFs expression is a predictor of poor prognosis, with further evidence demonstrating that a combination of sorafenib and HIFs-targeted therapy or HIFs inhibitors can overcome HCC sorafenib resistance. Here, we describe the molecular mechanism underlying sorafenib resistance in HCC patients, and highlight the impact of hypoxia microenvironment on sorafenib resistance.

## Introduction

The globally cancer statistics of 2018 show that liver cancer is the sixth most commonly diagnosed form of cancer, and a fourth cause of cancer-related deaths worldwide (1). Despite significant progress being made in development of therapies for early diagnosis and treatment therapies for HCC in recent years, over 50% of all HCC cases are still diagnosed at an advanced stage. Additionally, approximately 70% of all HCC patients relapse within five years of initial treatment (2). Current treatment options for HCC include radiotherapy, chemotherapy, local ablation and molecular targeted therapy (3). Several targeted inhibitors have also been developed and applied in clinical practice. For example, sorafenib, which acts as a multiple-target tyrosine kinase inhibitor (TKI), was the first systematic drug to be approved for advanced HCC patients based on results of two randomized clinical trials. Functionally, sorafenib inhibits proliferation and angiogenesis of tumor cells, thereby delaying HCC progression while effectively prolonging the survival time of patients (4). A previous Sorafenib Hepatocellular Carcinoma Assessment Randomized Protocol (SHARP) trial confirmed that the drug was safe and efficacious in patients with advanced HCC. In fact, these similar results were corroborated by findings in Asia-Pacific clinical trials (5, 6), in which sorafenib improved the clinical symptoms of about 30% of HCC patients. However, this group of patients reportedly develop resistance to sorafenib within 6 months of treatment (7). Previous studies indicate that sorafenib inhibits activity of various kinases, including Ras, Raf, MEK, and ERK, among others, and further targets VEGFR, c-KIT, PDGFR-β, and FLT-3, as well as other proteins that suppress tumor angiogenesis (8, 9). Moreover, sorafenib plays an anti-tumor role in HCC and other types of cancer, such as desmoid tumor, renal cell carcinoma, lung cancer and thyroid cancer (10–13). Although the drug effectively prolongs survival rates of HCC patients, its efficacy is significantly limited by development of drug-acquired resistance. The underlying mechanism of sorafenib resistance is complex. Previous studies have shown that the drug activates c-Jun, Akt pathway, epidermal growth factor receptor (EGFR), cancer stem cells enrichment, epithelial-mesenchymal transition (EMT) enhancement and reduces autophagy. Recently, other factors, such as dysregulation of miRNAs and lncRNAs in HCC have been implicated in sorafenib resistance (9, 14). Moreover, sorafenib reportedly induces hypoxia response in HCC, with dysregulation of hypoxia microenvironment and HIF expression shown to contribute to poor prognosis of HCC patients. In addition, sorafenib has also been implicated in effective inhibition of the HIF-1α/VEGFA signaling pathway (15). Weinberg et al. described six hallmarks of cancer, namely evasion of growth suppression, sustained proliferative signaling, induction of angiogenesis, resistance to cell death, replicative immortality, as well as activation of invasion and metastasis, and further demonstrated that these biological behaviors influence the degree of malignancy (16). Hypoxia, a common event that plays important roles in development and progression of malignant tumors, has been implicated in development of drug resistance and activation of tumor metastasis (17, 18). In the present study, we sought to clarify the underlying mechanism of sorafenib resistance, its relationship with the hypoxia microenvironment and the effect of targeting HIFs on sorafenib resistance in hepatocellular carcinoma.

## Sorafenib Resistance In Hcc

**Figure 1:**
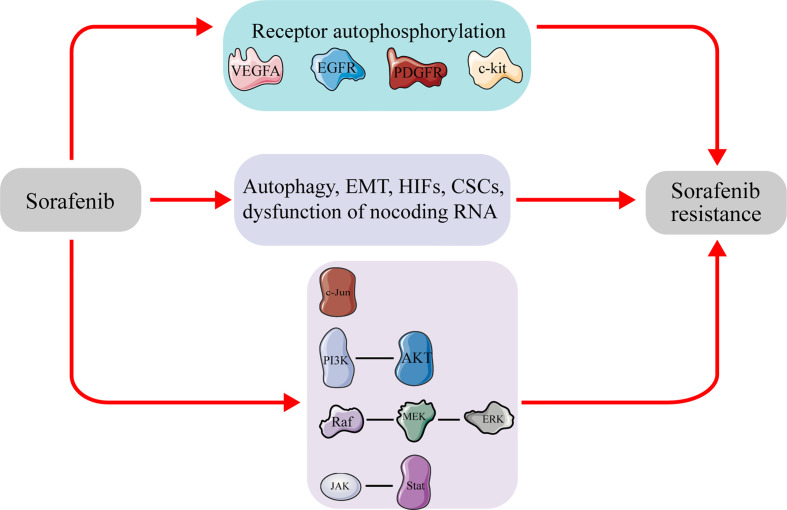
Molecules and signal pathways related to sorafenib resistance in hepatocellular carcinoma. Sustained sorafenib treatment will affect the expression of the molecules and activate pathways, leading to sorafenib resistance in hepatocellular carcinoma. VEGFA, vascular endothelial growth factor A; EGFR, epidermal growth factor receptor; PDGFR, Platelet-derived growth factor receptor; c-kit, tyrosine kinase receptors hepatocyte factor receptor; EMT, epithelial–mesenchymal transition; CSCs, cancer stem cells; PI3K, phosphatidylinositol-3-kinase; AKT, protein kinase B; ERK, extracellular signal-regulated kinase; JAK, janus tyrosine kinase; STAT, signal transducer and activator of transcription.

Drug resistance is divided into the primary and acquired resistance, based on the time and sequence of exposure to the drug. Although both categories involve a complex chemical resistance mechanism, signaling pathways, characterized by up-/down-regulation and changes in molecular targets, represent the two most important factors (19). Elucidating the underlying mechanism of drug resistance is imperative to development of effective strategies to prevent or overcome its development (**Figure 1**).

c-Jun also known as AP-1 transcription factor subunit. It’s located at 1p32-p31, Deletion and translocation of this chromosomal region has been associated with development of malignant tumors. A previous study reported that regulation of mitotic signals can activate AP-1 (20). while others have shown demonstrated its significance in hepatocyte activity and liver regeneration, as well as in development of hepatocellular carcinoma (21). Previous studies have also shown that c-Jun was remarkably activated in sorafenib-treated HCC cells, with its downregulation found to significantly elevate apoptosis of HCC cells induced by sorafenib (22). Another *in vitro* study found that sorafenib treatment could activate expression of c-Jun, while its inhibition significantly enhanced sorafenib-induced apoptosis in HCC cells (23). Results from a clinical trial revealed that HCC patients approached with sorafenib, the expression of Phosphorylation C-Jun was remarkably higher in the non-responder group than in the responder group (24). Therefore, c-Jun is probably one of the molecules that causes of HCC resistance to sorafenib.

The PI3K/AKT signaling pathway plays a role in regulation of apoptosis and chemotherapeutic resistance in malignant tumors. Previous studies have shown that sorafenib-mediated inhibition of AKT expression enhanced apoptosis induction in HCC cells (25). Moreover, Zhang et al. found that long term exposure of HCC cells to sorafenib could activate the PI3K/Akt signaling pathway, thereby whereas inhibiting PI3K using LY294002 could reverse sorafenib resistance (26). In another study, sorafenib effectively promoted AKT phosphorylation but did not significantly affect that of other proteins in the PI3K/AKT/mTOR signaling pathway (27). Therefore, activation of the PI3K/AKT signaling pathway is considered a compensatory mechanism for acquired sorafenib resistance. In fact, numerous studies have demonstrated that HCC cells with acquired sorafenib resistance exhibit significantly higher levels of phosphorylation AKT than parental cells, although suppression of AKT can reverse acquired sorafenib resistance (27, 28). The activation of AKT pathway has an important impact in sorafenib resistance in HCC.

Epidermal growth factor receptor (EGFR), which belongs to the protein kinase superfamily, can induce receptor dimerization and regulate autophosphorylation of tyrosine thereby causing cell proliferation. This phenomenon has been shown to be a potential indicator of sorafenib resistance in HCC cells. In HCC cells with higher EGFR expression, the efficacy of sorafenib is significantly weakened. A previous study demonstrated that sensitivity of cells to sorafenib could be increased by downregulating EGFR expression or inhibiting its kinase activity (29). In, Moreover, the EGFR pathway is overexpressed in HCC cells with acquired resistance to sorafenib, where it acts as the driving force for maintaining HCC cell proliferation under sorafenib (30).

Epithelial–mesenchymal transformation (EMT) contributes to migration and drug resistance, and is therefore an important cellular program (31). Previous studies have reported that EMT is associated with cancer chemotherapeutic resistance, with its inhibition found to reverse this drug-resistant outcome (32). For example, Fisher et al. established a genetically engineered mouse model and demonstrated the relationship between cancer drug resistance and EMT (33). Moreover, induction of EMT reportedly promotes tumor progression and sorafenib resistance in HCC. Although EMT development is inhibited by sorafenib, it has also been reported to promote chemotherapy resistance to sorafenib in HCC cells (34, 35). Previous studies have demonstrated that cancer stem cells (CSCs) are also involved in development of chemoresistance in HCC. In addition, there is crosstalk between EMT and CSCs, as evidenced by the fact that acquired EMT cells exhibit CSCs-like characteristics, while CSCs show mesenchymal phenotype (36), EMT activation is usually associated with enrichment of CSCs subsets in sorafenib resistance cells (37, 38). Thus CSCs markers have been used as predictors of sorafenib reaction. Notably, upregulation of CSC markers, CD90 and CD133, was shown to be a predictor for sorafenib resistance in HCC cells. Moreover, PTK2 activated CSC characteristics to promote tumor progression by inducing β-catenin nuclear accumulation in HCC cells thereby inducing sorafenib resistance (39). CD44 can be used as a marker for evaluating efficacy of sorafenib in HCC cells, as evidenced by its role in development of drug resistance (40). Other markers, such as CD13, EpCAM, and CD24 are also considered helpful for CSCs enrichment in HCC (41).

Autophagy, a self-degrading system that directs cells to eliminate abnormal proteins and dysfunctional organelles, plays an essential role in maintaining homeostasis in cells under stress, such as nutritional deficiency or hypoxia (42). In fact, autophagy plays a double-edged sword in different cancers by suppressing initiation of tumors but also supporting their progression. This mechanism further plays an important role in drug resistance, enabling tumor cells to maintain cell activity under metabolic and therapeutic stress. In fact, autophagy is often activated in radiotherapy, chemotherapy, and targeted therapy (43). In HCC, elevated autophagy reportedly regulated sorafenib resistance (44). Patients treated with sorafenib were found to overexpress Atg7 and had elevated autophagy activity, indicative of poor prognosis (45). However, another study demonstrated that sorafenib induced autophagy and further enhanced the drug’s effect on HCC, contrary to previous results (46). Furthermore, different HCC cell lines showed varied sensitivities to sorafenib, possibly due autophagy (47). The mechanism of autophagy has not been fully elucidated, and further research in this field is worthy of further study.

Numerous studies have reported that non-coding genes can also play an important role in development of chemotherapy resistance in cancers. For example, some miRNAs associated with sorafenib resistance have been identified, and can be used as biomarkers for predicting sorafenib treatment in HCC (**Table 1**).

**Table 1:** miRNA and sorafenib resistance in HCC cells.

Name	Cell line/animal models	Target	Reference
miR-21↑	HepG2, Huh7/BALB/c nude mice subcutaneous HCC model	AKT↑	(48)
miR-16↓	Huh7/BALB/c nude mice subcutaneous HCC model	14-3-3η↑,HIF-1α↑	(49)
miR-494↑	Huh7, SNU182, HepG2/DEN-treated rats	AKT↑, mTOR↑, P27↓, PUMA↓	(50)
miR-221↑	HepG2, Hep3B, PLC/PRF/5, SNU398, SNU449, SNU182, SNU475, Huh7/DEN-treated rats, NOD/SCID mice hydrodynamic tail vein injection	Caspase-3↓	(51)
miR-222↑	HepG2, HL-7702/–	AKT↑	(52)
miR-223↑	Huh7, SNU387, SNU449/–	FBW7↓	(53)
miR-622↓	PLC, Hep3B, HepG2, Huh7/male mice orthotopic tumor injected with HCC cells	KRAS↑	(54)
miR-347b↓	Hep3B, HepG2, HCCLM3/male SCID mice subcutaneous HCC model	PKM2↑	(55)
miR-181a↑	Hep3B, HepG2/–	RASSF1↓	(56)
miR-122↓	Huh7, PLC, T1115/NOD/SCID mice subcutaneous HCC model	IGF-1R↑	(57)
miR-122↓	HepG2, Hep3B, Huh7/DEN-HCC rat	SerpinB3↑	(58)
miR-744↓	LO2, HepG2, MMC-7721/–	PAX2↑	(59)
miR-137↓	Huh7/–	ANT2↑	(60)
miR-7↓	Huh7, Hep3B/mice orthotopic liver cancer model and tail vein injection	TYRO3↑	(61)
miR-142-3p↓	HepG2, SMMC-7721/BALB/c nude mice subcutaneous HCC model	PU.1↓ATG5↑ATG16L1↑	(62)
miR-3163↓	MHCC97-H, LM-3, HepG2, Hu7, BEL-7402, SMMC-7722, MHCC97-L/nude mice subcutaneous HCC model and tail vein injection	ADAM-17↑	(63)
miR-140-3p↓	MHCC97-H, HepG2/nude mice subcutaneous HCC model and hepatic portal vein injection	PXR↑	(64)
miR-30e-3p↓	HepG2, Hep3B, Huh7, SNU449, SNU475/DEN-HCC rat	MDM2↓ TP53↑	(65)
miR-19a-3p↑	PLC/PRF/5, BEL-7402, Hep3B and HepG2/–	PTEN↓	(66)
miR-486-3p↓	SK-HEP-1, HepG2, Huh7/BALB/C nude mice orthotopic HCC model and Subcutaneous HCC model	FGFR4↑ EGFR↑	(67)
miR-591↓	HepG2, Hep3B, SK-HEP1, HUH7/BALB/c nude mice subcutaneous HCC model	FBP2↑ AKT↑	(68)
miR-194↓	HUH7, HCCLM3/NOD-SCID mice subcutaneous HCC model	RAC1↑	(69)
miR-613↓	Huh7, HCCLM3/NOD-SCID mice subcutaneous HCC model	SOX9↑	(70)
miR-365↓	HCCLM3, SMMC7721/–	RAC1↑	(71)
miR-29a↓	Huh7, HepG2/NOD-SCID mice subcutaneous HCC model	BCL-2↑	(72)
miR-34a↓	Huh-7, MHCC97H/–	BCL-2↑	(73)
miR-219↑	HCCLM3, HepG2/NOD-SCID mice subcutaneous HCC model	E-cadherin↓	(74)
miR-216a/217↑	HepG2, Hep3B, Huh-7, PLC/PRF/5, HCCLM3, Bel-7404, HLE, SK-HEP-1, SNU-449/BALB/c nude mice orthotopic tumor injected with HCC cells	PTEN↓ SMAD7↓	(75)
miR-378a-3p↓	Huh7, HCCLM3, SK-HEP-1/BALB/C nude mice orthotopic HCC model, NOD/SCID mouse subcutaneous HCC model	IGF-1R↑	(76)
miR-522↑	Huh7, HCCLM3/NOD-SCID mice subcutaneous HCC model	PTEN↓	(77)
miR-494↑	Huh7, HepG2/–	PTEN↓	(78)
miR-375↑	Hep3B, HepG2, Huh1, Huh7/BALB/C nude mice subcutaneous HCC model	AEG-1↓PDGFC↓	(79)
miR-338-3p↓	HepG2, SMMC-7721, BEK-7402, Hep3B, Huh-7/BALB/c nude mice subcutaneous HCC model	HIF-1α↑	(80)
let-7↓	Huh7, HepG2/–	Bcl-xL↑	(81)
miR-193b↓	HepG2 and HepG2.2.15 (derived from HepG2 cells and stably integrated with the entire HBV genome)/	Mcl-1↑ (HBV infection induce sorafenib resistance)	(82)

miRNAs play various functions like mediate proliferation, invasion and metastasis, angiogenesis, induction of hypoxia, and et al. For example, low expression of some miRNAs had been found to promote sorafenib-resistance of HCC cells, miRNAs although others may have an opposite effect. A previous study reported that miR-486-3p inhibits cell proliferation and induces apoptosis, however, it was downregulated in sorafenib-resistant HCC cell lines by up-regulating FGFR4 and EGFR activity (67). In contrast, miR-216a/217 cluster was significantly upregulated in HCC compared to normal cells. although this upregulation could activate the TGF-β and PI3K/AKT signaling pathways, thereby contributing to acquired sorafenib resistance in HCC cells (75). miRNAs functional mechanism is complicated and still controversial. Different miRNAs have different effects on HCC, moreover, the same miRNA could have different effects on different cancers. There is still plenty of room for research in this field. Apart from miRNAs, many lncRNAs have also been implicated in sorafenib resistance (**Table 2**).

**Table 2:** lncRNA and sorafenib resistance in HCC cells.

Name	Cell line/animal models	Target	Reference
SNHG1↑	HepG2, Huh7/BALB/c nude mice subcutaneous HCC model	AKT↑	(83)
SHNG3↑	PLC/PRF/5, Hep3B, HepG2, MHCC97L, Huh7, SMMC-7721, HCCLM3/–	EMT↑	(84)
SHNG16↑	HepG2, SK-hep1, Huh7, HCCLM3, LO2/nude mice subcutaneous HCC model	–	(85)
FOXD2-AS1↓	HepG2, Huh7/–	TMEM9↓	(86)
NEAT1↑	HepG2, Huh7/–	ATG3↑	(87)
DANCR↑	HEK-293T, Huh7, Hep3B/BALB/c nude mice subcutaneous HCC model	STAT3↑	(88)
HOTAIR↑	Huh7, Hep3B, SNU-387, SNU-449/–	EMT↑	(89)
HEIH↑	Huh7, HCCLM3/–	AKT↑	(90)
MALAT1↑	HepG2, SMMC-7721/nude mice subcutaneous HCC model and tail veins injection	Aurora-A↑	(91)
ROR↑	LO2, HepG2, SMMC-7721, Huh7, MHCC97H, Hep3B, HCCLM3/BALB/c nude mice subcutaneous HCC model	FOXM1↑	(92)
Thor↑	HCCLM3, SMMC7721/	β-catenin↑	(93)
Ad5-A↓	HepG2, Huh7/BALB/c nude mice subcutaneous HCC model	AKT↑	(94)
HOXA13↑	SNU-449, HepG2/–	–	(95)
TUC338↑	HepG2, SMMC-7721, bEK-7402, Hep3B, Huh7, LO2/nude mice subcutaneous HCC model	RASAL1↓	(96)
HANR↑	HepG2, Huh7, 293T/BALB/c nude mice subcutaneous HCC model	ATG9A↑	(97)
H19↑	Huh7, Hep3B, SNU-449, SNU-387/–	EMT↑	(98)
H19↓	HepG2, Huh7, Plc/DEN-treat HCC mice model	–	(99)

Previous studies have reported that FOXD2-AS1 is downregulated in sorafenib-resistant HCC cells. Moreover, targeting FOXD2-AS1 was associated with inhibition of the NRf2 signaling pathway by regulating expression of TMEM9 and reversing resistance to sorafenib in HCC (86). Fan et al. found that MALAT1 was significantly up-regulated in sorafenib-resistant HCC cells, suggesting that it regulates Aurora A to promote cell proliferation, migration and EMT formation, thereby promoting the observed resistance (91). The expression levels of lncRNAs were significantly different in different tissues, and their functions were also different, the mechanisms that mediate the generation of functions are complex and diverse. LncRNA-mediated cell drug resistance is an emerging field, and in many current studies on lncRNA, their roles are also different.

## Hypoxic And Sorafenib-Resistance In Hcc

Hypoxia, which often occurs in many solid tumors, including HCC, is caused by faulty vascularization and vigorous metabolic activity, and has been associated with chemoresistance, increased invasiveness, and poor prognosis (100). Thus, suppressing hypoxia is considered a feasible approach for overcoming drug resistance. HIFs are transcription factors related to regulating angiogenesis, proliferation, glucose metabolism, tumor invasion and metastasis (100). Particularly, HIF-1α, -2α, -3α and -β are key factors that play a role in regulating a range of genes to control the hypoxia-induced signaling pathway. Since expression of the α-subunit is sensitive to oxygen, while the β-subunit is constitutively expressed, this review focuses on the α-subunit of HIFs (101). Among known α-subunits, HIF-1α and HIF-2α have been shown to regulate occurrence of hepatocellular carcinoma, while HIF-3α has generally been associated with inhibition of HIF-1α and HIF-2α activities (102, 103). Previous studies have shown that multiple factors are involved in hypoxia signal conversion from HIF-1α to HIF-2α (104). Moreover, adaptability of tumor cells to hypoxia changes with regulation of HIF-1α and HIF-2α. For example, hypoxia upregulates HIF-1α expression, and can cause it to bind to the hypoxia response element (HRE) of the target gene promoter, leading to transcription of related genes involved in hypoxia effects (105). HIF-1α is usually up-regulated in patients with liver cancer, with its overexpression associated with poor prognosis (106). Zhao et al. demonstrated that continuous sorafenib treatment could induce hypoxia and protect HCC cells to against the resulting apoptosis in HCC patients. During this process, HIF-1α was upregulated in untreated patients. Furthermore, HCC samples resistant to sorafenib exhibited HIF-1α levels above the sensitive groups (107).

Hypoxia not only activates HIF-1α in HCC, but also promotes production of VEGF and angiogenesis through HIF-1α activation (15). However, sorafenib reportedly suppresses HIF-1α synthesis, thereby causing a reduction in VEGF and tumor angiogenesis in HCC (108). Apart from that, suppression of sorafenib causes hypoxic response to be converted from HIF-1α to HIF-2α-dependent pathways, thereby promoting sorafenib resistance in hypoxic HCC cells. HIF-2α is also upregulated through a compensation mechanism, resulting in corresponding overexpression of VEGF and cyclin D1 (109).

The PI3K/AKT signaling pathway has also been widely associated with hypoxia-induced sorafenib resistance in HCC. Notably, several proteins have been shown to affect this pathway by reversing sorafenib induced hypoxia. Bort et al. reported downregulation of the AMPK/phosphorylated AMPK signaling pathway in sorafenib-resistant cells, with inhibition of this pathway *via* AMPK activation shown to affect sensitivities of HCC cells to sorafenib (110). Interestingly, down-regulation of AMPK also upregulates HIF-1α, and cooperates with c-myc to increase tumorigenesis, inducing and enhancing the CSCs of HCC cells, while its upregulation AMPK restores the sensitivity of HCC cells to sorafenib (111, 112). In addition, Yeh et al. showed that galectin-1 is elevated in sorafenib-resistant HCC cells, both *in vitro* and *in vivo*, promoting tumor metastasis and increasing tumor invasion, suggesting that galectin-1 plays a role in downstream regulation of the AKT/mTOR/HIF-1 signaling pathway (113). Hypoxia induces overexpression of AQP3 in hypoxic HCC cells, thereby altering sensitivity of these cells to sorafenib by activating the PI3K/Akt signaling pathway (114).

Other proteins have also been reported to alter resistance of HCC cells to sorafenib by acting on HIFs. For example, sorafenib was found to inhibit TIP30, thereby promoting EMT, which caused resistance to the drug (115, 116). Overexpression of HIF-2α was shown to downregulate TIP30 and promote EMT, although its down-regulation could reverse these effects (117). BNIP3 is also a hypoxic-regulated protein. Blanco et al. showed that HIFs stability and overexpression not only silenced the BNIP3 promoter, but also inhibited sorafenib-mediated apoptosis, thereby contributing to acquired drug resistance in HCC cells (118). On the other hand, RIT1, which belongs to the Ras superfamily, was shown to induce overexpression of RIT1 in HCC cells under HIF-1α-mediated hypoxia. Notably, sorafenib treatment could upregulate RIT1, while downregulating RIT1 in HCC cells could restore sensitivity of the cells to sorafenib (119). In addition, Long et al. found that PFKFB3 was upregulated in sorafenib-treated HCC cells. Notably, overexpressing PFKFB3 significantly enhanced sorafenib resistance in these cells by downregulating expression of apoptosis-related molecules, while blocking HIF-1α inhibited the enhancement of PFKFB3 (120). Apart from proteins, miRNAs have also been shown to play an important role in hypoxia. For example, 14-3-3η could stabilize HIF-1α and maintain resistance to sorafenib in HCC by inhibiting degradation of ubiquitin-proteasome-dependent protein, thereby maintaining CSCs. In addition, miR-16 was shown to reverse sorafenib resistance by inhibiting the 14-3-3η/HIF-1α/CSCs axis (49).

Taken together, the findings of these studies affirm the relationship between HIF expression disorder and sorafenib resistance, suggesting that hypoxia may significantly affect the therapeutic effect of sorafenib. Therefore, targeting these factors holds promise to future development of effective therapies to overcome drug resistance (**Table 3**).

**Table 3:** Hypoxia and sorafenib resistance in HCC cells.

HIFs after sorafenib treat in HCC	Cell line/animal models	Target	Reference
HIF-1α↑	HepG2, Huh7/–	AKT↑	(114)
HIF-1α↑	HepG2, SMMC-7721, BEK-7402, Hep3B, Huh-7/BALB/c nude mice subcutaneous HCC model	–	(80)
HIF-2α↑HIF-1α↓	HepG2, Huh7/BALB/c mice subcutaneous HCC model	VEGF↑cyclinD1↑LDHA↓	(109)
HIF-1α↑	–/Kunming mice subcutaneous HCC model	AKT↑	(121)
HIF-2α↑HIF-1α↓	HepG2, Bel-7402, Huh-7, SMMC-7402/BALB/c mice subcutaneous HCC model	PCNA↑β-catenin↑ C-Myc↑	(122)
HIF-1α↑	HepG2, Huh7/Athymic nude‐Foxn1 subcutaneous HCC model	AMPK↓AKT↑	(112)
HIF-1α↑	Huh7/BALB/c mice subcutaneous HCC model and tail vein inoculation model	Galectin↑mTOR↑	(113)
HIF-1α↑	Hep3B/–	mTOR↑	(123)
HIF-1α↑	LM3, SMMC-7721, Bel-7402, HepG2/nude mice subcutaneous HCC model	PPAR-γ↑PKM2↑	(124)
HIF-2α↑	MHCC97H/BALB/c mice subcutaneous HCC model and orthotopic model	TIP30↓EMT↑	(117)
HIF-1α↑	LM3, SMMC-7721, Hep3B, Bel-7402, Huh-7, LO2/BALB/c mice subcutaneous HCC model	GULT1↑HK2↑	(125)
HIF-1α↑	LM3, SMMC-7721, Bel-7402, Huh-7, HepG2, LO2/BALB/c mice subcutaneous HCC model	PKM2↑	(126)
HIF-2α↑HIF-1α↑	HepG2/–	BNIP3↓	(118)
HIF-1α↑	Huh7/BALB/c nude mice subcutaneous HCC model	14-3-3η↑	(49)
HIF-2α↑HIF-1α↑	HepG2, Hep3B, SK-Hep-1/BALB/c mice subcutaneous HCC model	ATPaseα3↑	(127)
HIF-2α↑	HepG2, SKhep1/BALB/c mice subcutaneous HCC model and tail vein inoculation model	androgen receptor↓	(128)
HIF-1α↑	HepG2, Hep3B, PLC/5, SK-Hep-1/BALB/c mice subcutaneous HCC model, tail vein inoculation model and orthotopic model	VEGF↑MDR1↑P-gp↑ GULT1↑NF-κB↑	(129)
HIF-2α↑HIF-1α↓	HepG2/BALB/c mice subcutaneous HCC model	TGF-α↑EGFR↑	(107)
HIF-1α↑	Hep3B, HepG2, PLC/PRF/5, HEK 293T/BALB/c mice orthotopic model	RIT1↑	(119)
HIF-1α↑	SK-Hep-1, SMMC-7721, HepG2, Huh7, MHCC-97H, LM3/–	PFKFB3↑	(120)

## Strategies To Overcome Sorafenib Resistance In Hcc By Target Hifs

**Figure 2:**
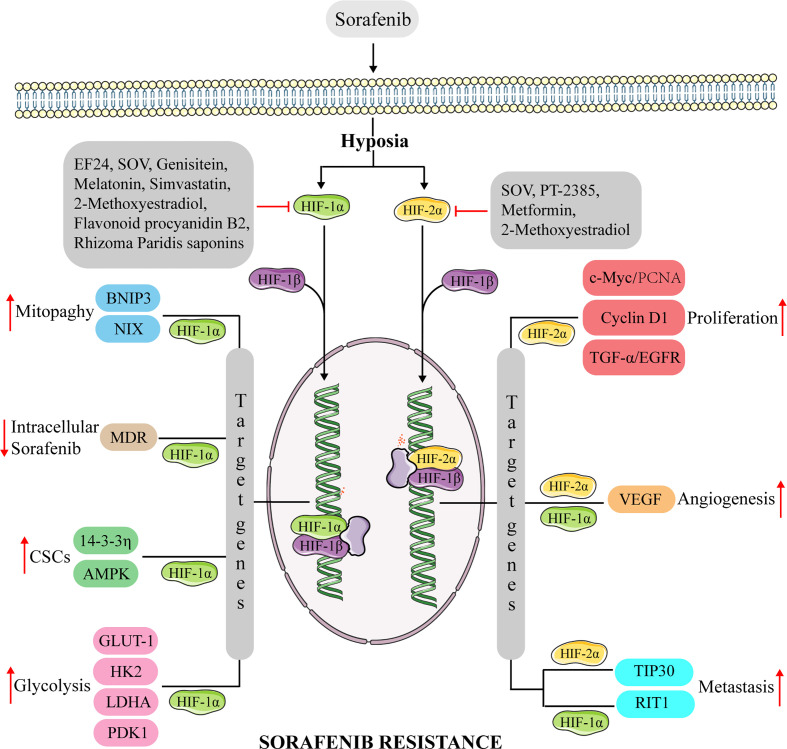
The mechanism of anti-HIFs overcoming sorafenib resistance in hepatocellular carcinoma. Continuous sorafenib treatment induce the dysregulation of HIF-1α and HIF-2α expression in hepatocellular carcinoma, promoting the transcription of multiple genes involved in proliferation, CSCs, metastasis, glycolysis, mitophagy, and angiogenesis. Causes hepatocellular carcinoma to develop resistance to sorafenib. Anti-HIFs could overcome this drug resistance. BNIP3, adenovirus E1B 19kDa-interacting protein 3; NIX, BNIP3-like protein X; MDR, multidrug resistance protein; AMPK, AMP-activated protein kinase; GULT-1, glucose transporter 1; HK2, hexokinase 2; LDHA, lactate dehydrogenase A; PDK1, pyruvate dehydrogenase kinase isoform 1; c-Myc, Myc proto-oncogene protein; TGF-α, transforming growth factor α; RIT1, Ras like without CAAX 1.

Considering that HIFs participate in a variety of cancer-promoting pathways and regulate the biological behavior of HCC cells, targeting HIFs may be an effective treatment strategy. For example, since HIFs play a key role in development of HCC resistance to chemotherapy drugs, inhibiting them could be a feasible strategy to manage drug resistance in HCC cells. Sustained sorafenib treatment leads to increased hypoxia in the tumor, thus targeting HIFs can enhance efficacy of sorafenib. Previous studies have shown that several drugs can reverse sorafenib-resistance in HCC by targeting HIF (**Figure 2**).

For example, sodium orthovanadate is a phosphate analogue that inhibits the cell cycle of sorafenib-resistant HCC cells by regulating cyclin B1 and CDK1 (arrest is in the G2/M phase). It also mediates a reduction in the mitochondrial membrane potential to induce apoptosis. A previous study demonstrated that sodium orthovanadate could down-regulate HIF-1α and HIF-2α, thereby causing a corresponding down-regulation of downstream molecules, such as VEGF, lactate dehydrogenase A and glucose transporter 1 (127). Similarly, melatonin was found to down-regulate HIF-1α protein synthesis by inhibiting the pathway of rapamycin complex-1/ribosomal protein S6 kinase β-1/ribosomal protein S6. In addition, co-administration of sorafenib and melatonin downregulated HIF-1α mitotic targets, NIX and BNIP3, thereby enhancing sensitivity of HCC cells to sorafenib (123). Notably, a combination of melatonin and sorafenib was shown to regulate the JNK/c-Jun signaling pathway, to synergistically suppress proliferation of HCC cells and induce apoptosis (130). Moreover, You et al. reported that a combination of metformin and sorafenib could synergistically inhibit HIF-2α expression, thereby increasing sensitivity of hypoxic HCC cells to sorafenib, and hindering EMT. This combination was also associated with inhibition of the growth of recurrent tumors and could significantly reduce the number of metastases *in vivo* (117). Feng et al. demonstrated that Simvastatin could suppress the HIF-1α/PPAR-γ/PKM2 signaling pathway by inhibiting PKM2-mediated glycolysis, thereby promoting and lowering apoptosis and proliferation of HCC cells, respectively. In addition, the drug also enhanced the effect of sorafenib in HCC cells (124). Another drug, 2-Methoxyestradiol, was shown to significantly downregulate HIF-1α and HIF-2α expression as well as that of downstream molecules such as VEGF, cyclin D1, and LDHA. Notably, its synergistic interaction with sorafenib reportedly inhibited proliferation of HCC cells and induced apoptosis both *in vivo* and *in vitro*, thereby inhibiting tumor angiogenesis (109).

Certain natural compounds have also shown efficacy in improving sorafenib-mediated treatment in drug-resistant liver cancer cells. For example, flavonoid procyanidin B2 was shown to downregulate PKM2 expression, thereby affecting the PKM2/HSP90/HIF-1α axis, inhibiting aerobic glycolysis, as well as proliferation and induction of apoptosis in HCC cells. Notably, co-treatment of procyanidin B2 and sorafenib could effectively improve the latter’s sensitivity in HCC cells (126). Genistein, a natural isoflavone that inhibits glycolysis, was shown to induce apoptosis and down-regulate GLUT-1 and HK2 by suppressing HIF-1α, thereby enhancing the effect of sorafenib on drug-resistant HCC cells both *in vitro* and *in vivo* (125). In addition, saponins derived from Rhizoma Paridis significantly downregulated mRNA expression and protein levels of HIF-1α, and further exhibited their anti-tumor activity by regulating glycolysis and lipid metabolism. Notably, a combination of these saponins with sorafenib could improve the anti-tumor effect *in vivo*. Previous studies have further shown that g sorafenib resistance in liver cancer cells can be overcome by preventing mitochondrial damage, inhibiting anaerobic glycolysis and suppressing lipid synthesis by targeting the PI3K/Akt/mTOR signaling pathway (121). For example, EF24 effectively reversed sorafenib resistance by degrading HIF-1α and inactivating NF-κB *via* a VHL tumor suppressor. A combination of EF24 with sorafenib was also found to generate a synergistic effect that enhanced the associated anti-tumor effect (129).

Previous studies have also reported that application of PT-2385 could specifically inhibit HIF-2α, to increase androgen receptors, suppress downstream factors such as STAT3, and activate the Akt and ERK signaling pathways, thereby improve sorafenib efficacy in HCC cells both *in vivo* and *in vitro* (128). In summary, inhibiting HIFs can effectively enhance the sensitivity of HCC cells to sorafenib and improve drug resistance.

## Conclusion And Discussion

Although sorafenib is a safe and effective therapy for treating advanced HCC, development of drug resistance has been shown to reduce its benefits. The underlying mechanism of this resistance is complex and currently remains unclear. Primary drug resistance can be explained by the genetic heterogeneity of HCC. Elucidating the underlying mechanism of acquired drug resistance is important in guiding development of approaches to overcome or delay its development. Previous studies have demonstrated that sorafenib-acquired resistance involves multiple mechanisms, including crosstalk in the PI3K/Akt, MAPK, JAK-STAT, ERK and HIF signaling pathways, abnormal expression of proteins, such as PDGFR-β, c-KIT, FLT-3, VEGFR, EGFR, as well as EMT, cancer stem cells, and autophagy, among others. Abnormal regulation of miRNAs and lncRNAs, as well as development of hypoxia in HCC also play an important role in inducing acquired resistance to sorafenib.

For patients with advanced liver cancer, who have been exposed to sorafenib for a long time, the drug’s anti-angiogenic effect is expected to cause a decrease in microvessel density and enhance tumor hypoxia. Consequently, this induces the HIF-mediated cell adaptation mechanism to the hypoxic microenvironment. In other tumors, extensive researches have been done using gene therapy to target HIFs or adding HIFs inhibitors to current therapies, with a view to improve its effectiveness. Particularly, overexpression of HIFs in liver cancer has been reported, with sorafenib found to promote HIF activity. Notably, a combination of sorafenib with other drugs, to lower the level of or directly target HIFs, has been proven to improve efficacy of sorafenib, suppress the proliferation and promote apoptosis of HCC cells, as well as reduce the number of metastases and tumor volume both *in vitro* and *in vivo*. For patients with advanced HCC, who have failed sorafenib treatment, several drugs, such as lenvatinib, regorafenib, cabozantinib, and ramucirumab, have been approved for second-line treatment. However, sorafenib remains the mainstay for treating advanced HCC (131). The importance of overcoming sorafenib resistance in HCC cells cannot be overemphasized. For this, targeting HIFs and improving the tumor hypoxic microenvironment hold promise for future development of therapies to manage sorafenib resistance and improve prognosis of patients with advanced HCC.

## Author Contributions

ZZ, QL, YL, and JZ conceived the project and wrote the manuscript. QZ, LH, ZX, YT, and ZS participated in data analysis. QX participated in discussion and language editing. DH reviewed the manuscript. All authors contributed to the article and approved the submitted version.

## Funding

This work was funded by the Co-construction of Provincial and Department Project (WKJ-ZJ-1919), the National Science and Technology Major Project for New Drug (No. 2017ZX09302003004), and the Key Research and Development Project of Zhejiang Science and Technology Department (2020C03008).

## Conflict of Interest

The authors declare that the research was conducted in the absence of any commercial or financial relationships that could be construed as a potential conflict of interest.
